# Exploring the versatile roles of the endocannabinoid system and phytocannabinoids in modulating bacterial infections

**DOI:** 10.1128/iai.00020-24

**Published:** 2024-05-22

**Authors:** Hailey Barker, Mariola J. Ferraro

**Affiliations:** 1 Microbiology and Cell Science Department, IFAS, University of Florida, Gainesville, Florida, USA; Department of Microbiology and Environmental Toxicology, University of California at Santa Cruz, Santa Cruz, California, USA

**Keywords:** endocannabinoid system, cannabinoid receptors, anandamide, 2-arachidonoylglycerol, host-pathogen interactions, innate immune cells, intracellular infections, phagocytosis, macrophage polarization, bacterial pathogens, polymicrobial sepsis, gut microbiota

## Abstract

The endocannabinoid system (ECS), initially identified for its role in maintaining homeostasis, particularly in regulating brain function, has evolved into a complex orchestrator influencing various physiological processes beyond its original association with the nervous system. Notably, an expanding body of evidence emphasizes the ECS’s crucial involvement in regulating immune responses. While the specific role of the ECS in bacterial infections remains under ongoing investigation, compelling indications suggest its active participation in host-pathogen interactions. Incorporating the ECS into the framework of bacterial pathogen infections introduces a layer of complexity to our understanding of its functions. While some studies propose the potential of cannabinoids to modulate bacterial function and immune responses, the outcomes inherently hinge on the specific infection and cannabinoid under consideration. Moreover, the bidirectional relationship between the ECS and the gut microbiota underscores the intricate interplay among diverse physiological processes. The ECS extends its influence far beyond its initial discovery, emerging as a promising therapeutic target across a spectrum of medical conditions, encompassing bacterial infections, dysbiosis, and sepsis. This review comprehensively explores the complex roles of the ECS in the modulation of bacteria, the host’s response to bacterial infections, and the dynamics of the microbiome. Special emphasis is placed on the roles of cannabinoid receptor types 1 and 2, whose signaling intricately influences immune cell function in microbe-host interactions.

## INTRODUCTION

The endocannabinoid system (ECS) stands as a neuromodulator network encompassing cannabinoid (CB) receptors, their ligands, endocannabinoids (eCBs), and the enzymes orchestrating eCB synthesis and breakdown. These endogenous CBs, often referred to as endocannabinoids (eCBs), emerge as bioactive lipids forged from polyunsaturated fatty acids. Prominent among them are anandamide (AEA) (1) and 2-arachidonoylglycerol (2, 3), although other alternative eCBs have also merited investigation (4, 5). At the heart of this system’s functionality lies the binding of eCBs to the CB receptors— cannabinoid receptor 1 (CB_1_R) and cannabinoid receptor 2 (CB_2_R)—thus triggering its activation (6). This activation is not limited solely to endogenous compounds; it extends to plant-derived CBs and synthetic CBs, which also engage the same eCB system. The broad distribution of CB receptors and the capacity of CBs to influence cellular behavior have galvanized research across a spectrum of domains, encompassing mental health conditions, diabetes, fertility, and autoimmune disorders (7). Given the diverse cellular pathways influenced by the eCB system, it is conceivable that this network is implicated in host-pathogen interactions in bacterial infections, and several studies do suggest that this is the case.

This review aims to provide an overview of the effects exerted by eCBs, synthetic CBs, and plant-derived CBs on the innate immunity and relationship between host and microbe. Through this exploration, we aim to shed light on the potential impact of the eCB system within this dynamic context.

## THE ENDOCANNABINOID SYSTEM

The most important components of ECS are the endocannabinoids, the enzymes responsible for the synthesis and degradation of the eCBs, and the receptors that mediate the effects of the eCBs. However, apart from the eCBs, exogenous cannabinoids such as phytocannabinoids or synthetic cannabinoids can also interact with the ECS.

### Cannabinoids

Cannabinoids can be divided into different groups depending on the criterium used. Based on their origin, cannabinoids can be classified into three groups: phytocannabinoids, endocannabinoids, and synthetic cannabinoids. In terms of structure, cannabinoids are divided into four groups: classical cannabinoids, nonclassical cannabinoids, aminoalkylindoles, and eicosanoids, where the last group is represented by the two most important eCBs produced endogenously. Classical cannabinoids are dibenzopyran derivatives, such as phytocannabinoid Δ9-tetrahydrocannabinol (Δ9-THC) and more potent synthetic cannabinoid HU-210 (8). Nonclassical cannabinoids contain bicyclic and tricyclic analogs of Δ9-THC that lack a pyran ring, like CP 55,940 (9) or CP 47,497 (10). Next, aminoalkylindoles (AAI) have structures that differ from those of both classical and nonclassical cannabinoids. Subdivided into naphthoylindoles, phenylacetylindoles, benzoylindoles, and naphthylmethylindoles, these compounds became prevalent in smoking mixtures and are known as “Spice” (11). Aminoalkylindoles are well represented by WIN 55,212-2, a derivative of pravadoline. Compared with HU-210 and CP55,940, WIN 55,212-2 has been found to have a slightly higher affinity to CB_2_R than CB_1_R.

Finally, eicosanoids are derivatives of long-chain fatty acids within the eicosanoid superfamily and activate cannabinoid receptors. Key examples of arachidonic acid-based cannabinoids include anandamide (1), 2-arachidonylglycerol (2-AG) (2, 3), and virodhamine (12). Rapidly produced from lipid precursors, these eicosanoids are released in response to neuronal activity and pro-inflammatory agents (13). Specific triggers, like intracellular calcium-induced membrane depolarization and G-protein activation, initiate cascades culminating in distinct eCB generation and associated enzymes (14). While the synthesis of AEA and 2-AG has been extensively reviewed elsewhere (15–17) and will not be reviewed here in detail, select reactions leading to their synthesis are mentioned here. AEA synthesis involves at least five metabolic pathways (18). Despite similarities between mouse and human cell models, differences in enzymes required for eCB metabolism exist, exemplified by the presence of monoacylglycerol lipase (MGLL) enzymes in monocyte-derived macrophages but not in mouse bone marrow-derived macrophages (19). The main pathway for AEA production begins with the fatty acyl chain transfer from an N-acyltransferase (NAT) to produce N-acylphosphatidylethanolamine (NAPE) (18, 20). NAPE is further converted into AEA through the function of N-acyl-phosphatidylethanolamine-hydrolyzing phospholipase D (NAPE-PLD) (21). Three additional routes of AEA biosynthesis also involve the conversion of NAPE. These two pathways are quite similar: they use either α,β-Hydrolase-4 (ABHD4) or phospholipase A2 (PLA_2_) to produce the intermediate lyso-NAPE. Lyso-NAPE is then converted into AEA by lyso-phospholipase D (lyso-PLD) function in both pathways (22, 23). The last pathway that uses NAPE is the phospholipase C (PLC) pathway, in which PLC converts NAPE into P-AEA. Once the p-AEA precursor is generated, a protein tyrosine phosphatase (PTPN22) further converts p-AEA into the final product of AEA (24).

**Fig 1:**
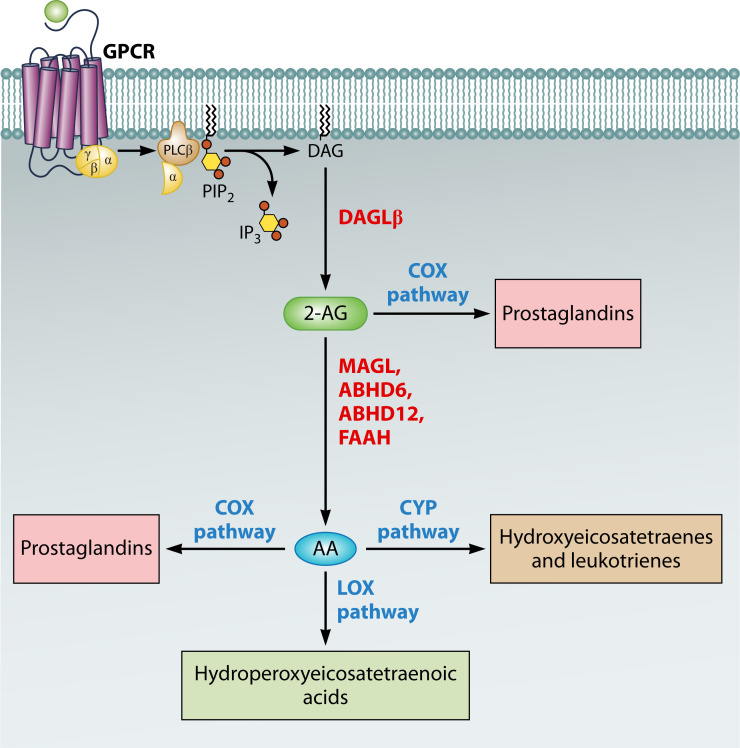
Representation of the biosynthesis and degradation pathways of 2-AG. This diagram illustrates the metabolic pathways involved in the synthesis and breakdown of 2-arachidonoylglycerol (2-AG). Stimulation of G protein-coupled receptors (GPCR) activates Phospholipase C (PLC), leading to the release of diacylglycerol (DAG) and inositol triphosphate. DAG is subsequently converted into 2-AG. The degradation of 2-AG is mediated by several enzymes: monoacylglycerol lipase (MAGL), alpha/beta-hydrolase domain 6 (ABHD6), alpha/beta-hydrolase domain 12 (ABHD12), and fatty acid amide hydrolase (FAAH), resulting in the production of arachidonic acid (AA). AA serves as a precursor for the synthesis of various eicosanoids through the cyclooxygenase (COX), lipoxygenase (LOX), and cytochrome P450 oxidase (CYP) pathways. These pathways generate bioactive lipids, such as prostaglandins, leukotrienes, and hydroperoxyeicosatetraenoic acids, which are critical for the inflammatory response and cellular signaling. Additionally, COX enzymes can directly transform 2-AG into prostaglandins.

The synthesis of 2-AG is a relatively more straightforward process than the production of AEA (Fig. 1). Currently, there are at least three main routes known for 2-AG synthesis. The primary pathway involves the activation of phospholipase Cβ through G protein-coupled receptor (GPCR) signaling. This activation leads to the hydrolysis of phosphatidylinositol 4,5-bisphosphate (PIP2) stored in the plasma membrane, producing diacylglycerol (DAG). Subsequently, diacylglycerol lipases (DAGL) α or β (predominantly expressed in neurons and immune cells, respectively) further hydrolyze DAG to generate 2-AG (15, 25, 26). The specific isoforms of phospholipase Cβ and Gq/11-coupled receptor pairs present in different cell types can give rise to variations in this biosynthesis pathway (27). Another less common pathway for 2-AG biosynthesis involves the enzymatic activity of 2-lysophosphatidic acid (LPA) phosphatase. When 2-AG is produced, it can be converted into 2-arachidonoyl-LPA by MAG kinase. However, researchers have observed that a significant portion of 2-arachidonoyl-LPA is converted back into 2-AG. This discovery led to the understanding that the function of MAG kinase can be reversed by 2-LPA phosphatase, thereby recreating 2-AG (28). Additionally, there is an alternative pathway in which phosphatidylinositol 3,4,5-trisphosphate (PIP3) is converted into phosphatidylinositol (PI), which is then utilized to generate 2-arachidonoyl-LPI through the action of phospholipase A1 (PLA1). 2-Arachidonoyl-lysophosphatidylinositol (LPI), derived from this pathway, functions as a bioactive lysophospholipid mediator, activating GPR55, p38 MAP kinase, and other signaling cascades (29–31). While phospholipase Cβ is the primary signaling mechanism responsible for eCB production, recent studies suggest the existence of alternative mechanisms to activate these production pathways. For instance, investigations have revealed that phospholipase Cγ2 can trigger the synthesis of DAG and 2-AG by activating the Fcγ receptor in immune cells (19).

The degradation of endocannabinoids, including 2-arachidonoylglycerol (Fig. 1) and anandamide, involves complex pathways leading to the production of arachidonic acid. The enzymatic breakdown of 2-AG is primarily facilitated by monoacylglycerol lipase, while fatty acid amide hydrolase is essential for the inactivation of AEA, highlighting the specificity of their catabolic routes (32, 33), although FAAH might also contribute in the hydrolysis of 2-AG (34). Additionally, ABHD6, a serine hydrolase, contributes to the hydrolysis of 2-AG, underscoring the diversity of enzymes involved in eCB metabolism (35, 36). Another significant enzyme, ABHD12, also a serine hydrolase but with predominant expression in microglia and brain tissues, plays a role in 2-AG metabolism, indicating the tissue-specific aspects of eCB regulation (37). Chemical modification of 2-AG and AEA involves the action of lipoxygenases, cyclooxygenases, and cytochrome P450 enzymes (CYP450), highlighting the complex nature of eCB catabolism. Various LOX isoforms, including LOX-5, 8-LOX, 11-LOX, LOX-12, and LOX-15, have been identified as participants in the breakdown of AA and potentially direct metabolization of 2-AG and AEA, pointing to the involvement of these enzymes in broader lipid signaling pathways (15, 38–41). COX-2 can oxygenate AEA and 2-AG, leading to the creation of an array of prostaglandin glycerol esters and ethanolamides. These metabolites have significant implications for inflammatory responses and cellular signaling mechanisms (42, 43). Lastly, the CYP450 enzyme family, specifically through its monooxygenase activity, is known to oxygenate AEA, producing various ethanolamides, which further illustrates the specificity and complexity of eCB metabolism (44, 45).

### Cannabinoid receptors

The term “cannabinoid” typically signifies the impact of these compounds on cannabinoid receptors. Here, two main receptors are of particular importance: CB_1_R) and CB_2_R, encoded by *cnr1* and *cnr2* genes, respectively. CB_1_R and CB_2_R are G-protein-coupled receptors that share approximately 44% homology, and the ligand binding domains have about 68% homology (46). The CB receptors are present in a wide range of cell types and are involved in various signaling pathways, although their expression varies in different tissues, depending on the receptor. CB_1_R is distributed throughout the central nervous system (CNS) and peripheral nervous system, particularly in axon terminals in regions such as the cerebellum, hippocampus, basal ganglia, frontal cortex, amygdala, hypothalamus, and midbrain (47, 48). CB_1_R is also present in hepatocytes, glucagon-containing α-cells, and adipocytes (49–51). Given the high expression of CB_1_R in neuronal cells, much of the current research focuses on understanding the relationship between CB_1_R activation and the modulation of biological systems in the CNS. The activation of this receptor by Δ9-THC, the principal active compound in *Cannabis sativa*, (52) causes the psychoactive effects of plant-derived CBs (53). CB_1_R also exhibits an affinity for synthetic cannabimimetic compounds such as CP55940, JWH-015, WIN 55212-2, and endocannabinoids (AEA and 2-AG) (54). Upon interaction with these ligands and CB_1_R couples with pertussis toxin-sensitive Gi/o type G proteins, initiating a rapid reduction in cyclic AMP (cAMP) levels by inhibiting adenylate cyclase activity (55–57). In addition to cAMP modulation, CB_1_R can deactivate A-type K+ channels and attenuate transmitter release. These effects are achieved through the direct interaction of the β/γ-subunit of the G protein with calcium channels in the neuronal pre-synaptic membrane (58). The complexity of CB_1_R signaling is further compounded by evidence of promiscuous coupling to various G proteins, the participation of β-arrestins in signaling, and signaling processes originating from intracellular compartments (59). Consequently, CB_1_R, like many other G protein-coupled receptors (GPCRs), exhibits a complicated nature. Moreover, CB_1_R displays constitutive activity, indicating G protein activation even in the absence of agonists (60). Inverse agonists such as SR141716A (also known as rimonabant), previously used as an anti-obesity treatment, can reverse this constitutive activity (61). The diversity in pharmacological interactions between cannabinoids and the CB_1_R and CB_2_R can be further emphasized by the fact that some cannabinoids function as full agonists of the cannabinoid receptors, such as WIN55,212-2, while others, such as Δ9-THC function as partial agonists (62), which can yield significantly different cellular responses and physiological outcomes. This complexity is compounded by the differences in the binding affinity of each cannabinoid for each CB receptor, adding another layer of complexity to CB_1_ R signaling.

In contrast to CB_1_R, CB_2_R exhibits a more distinct pattern of expression within the brain, primarily inhabiting cells and tissues associated with the immune system and concentrated predominantly in microglia, the resident macrophages of the CNS (63). However, CB_2_R is also present in immune cells outside of brain regions, for instance in monocytes, macrophages, B-lymphocytes, and T-lymphocytes (46, 64), suggesting a potential association of the ECS with immunomodulation (65–67). The high expression of the CB_2_R among immune cells and its role in maintaining immune homeostasis makes it a potential therapeutic target for immunological diseases and infections. Although CB_2_R does not couple to potassium channels like CB_1_R does, the stimulation of CB_1_R and CB_2_R leads to the activation of the p42/p44 MAPK (ERK1/2) pathway, responsible for cellular growth and proliferation, as well as the p38 MAPK pathway, governing cellular differentiation and inflammatory responses (55–57).

Apart from CB_1_R and CB_2_R, CBs can also interact with other receptors, such as ligand-sensitive ion channel receptors [transient receptor potential (TRP) channels], G-protein coupled receptors, and nuclear receptors, specifically Peroxisome proliferator-activated receptors (PPARs) (68). Six transient receptor potential (TRP) channels, namely, TRPV1, TRPV2, TRPV3, TRPV4, TRPA1, and TRPM8, have been identified as being modulated by eCBs, plant-derived CBs, or synthetic CBs (69). These six TRPs, known as ionotropic CB receptors, are now recognized for their role in the ECS (70). Transient receptor potential vanilloid 1 (TRPV1) was the first TRP channel discovered that could be activated by eCBs, specifically anandamide (71). TRPV1 is expressed alongside CB1R and CB2R in certain cell types, suggesting potential cooperation in regulating signaling cascades (72). PPARs are nuclear receptor proteins found in cells throughout the body responsible for regulating gene expression by acting as transcription factors to modulate cellular functions, including immune responses [reviewed in reference (73)]. Current research on CB interaction with PPARs suggests that eCBs, synthetic CBs, plant-derived CBs, and CB-like derivatives can activate various PPARs, primarily PPARα or PPARγ, leading to various physiological outcomes (74). Moreover, G protein-coupled receptor 55, primarily expressed in large dorsal root ganglion neurons, gets activated by CBs. Existing evidence indicates that GPR-55 can be triggered by eCBs, synthetic cannabinoids, and those derived from plants. Its activation involves pathways distinct from CB_1_R and CB_2_R, leading to an increase in intracellular calcium levels (75). CBs may also alter signaling pathways through allosteric binding to receptors such as opioid receptors, serotonin (5-HT)3 receptors, N-methyl-d-aspartate (NMDA) receptors, and nicotinic acetylcholine receptors (76).

## IMPACT OF CANNABINOID SIGNALING ON MACROPHAGE FUNCTION

**Fig 2:**
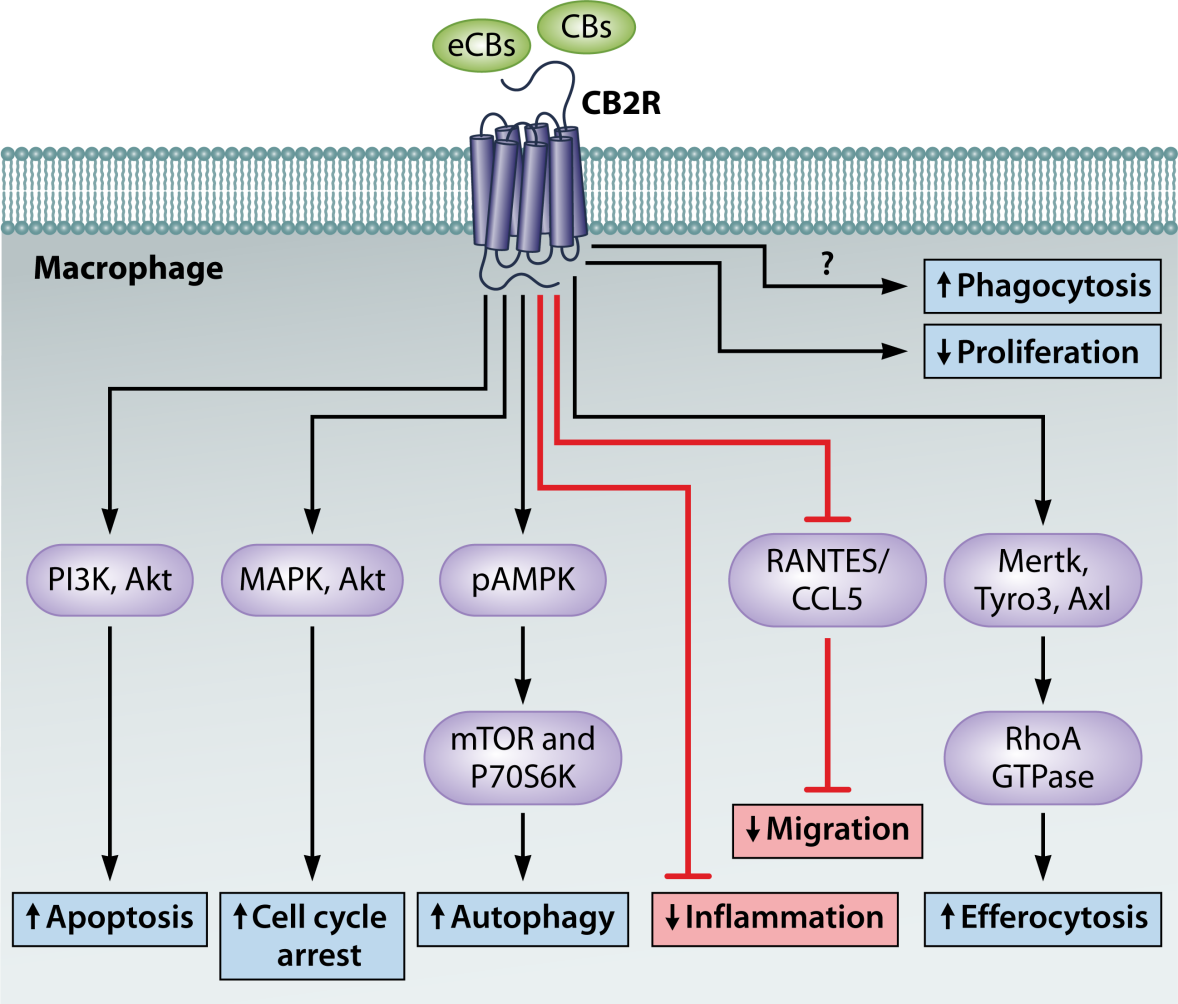
Most prominent cellular effects of CB_2_R activation. An overview of the cellular responses elicited by CB_2_R activation, including the activation of MAPK pathways (ERK1/2, P38) important for cell growth and inflammatory responses, autophagy induction for cellular homeostasis, apoptosis in immune cells, modulation of phagocytosis, and promotion of M2 macrophage polarization, illustrating CB_2_R’s role in immune regulation and its potential for treating inflammation-related conditions.

The impact of cannabinoid signaling on innate immunity functions is a burgeoning area of research, with a particular focus on the expression of CB_2_R in immune cells [reviewed in reference (77, 78)]. While this review does not comprehensively cover all aspects of innate immune cell function, it is essential to note that the ECS affects critical processes such as immune cell migration (79–83), autophagy (84–88), and apoptosis (89–92) (Fig. 2). Although CB_2_R is prominently expressed in immune cells, the influence of CB_1_R should not be underestimated, but it does require further study since there is a scarce amount of information about this receptor’s influence over innate immunity. Here, we will briefly describe the physiological and functional changes orchestrated by the ECS in the innate immune regulation of key macrophage processes that facilitate the clearance of bacteria. This includes discussions on phagocytosis and macrophage polarization, crucial mechanisms promoting the effective elimination of bacteria.

### Effect of cannabinoid signaling on phagocytosis

CBs exert influence on the critical cellular process of phagocytosis, primarily executed by macrophages and dendritic cells. Recent studies have revealed the intricate role of CB_2_ receptors within BV-2 microglial cells, showcasing a tendency toward reduced phagocytic capacity upon activation by endogenous or synthetic CBs, modulating through the β-arrestin2/ERK1/2 and PI3K/AKT/GSK-3β signaling pathway (93). In contrast to this observed reduction, palmitoylethanolamide (PEA) has exhibited an opposing effect, enhancing the expression of CB_2_R and promoting phagocytic activity in microglia and macrophages. This suggests a potential therapeutic avenue for managing neuroinflammation in CNS disorders. Notably, PEA’s enhancement of phagocytosis, specifically demonstrated in the engulfing of *Porphyromonas gingivalis* by microglial cells, was reversed when the CB_2_R was blocked using AM630 (94). Similarly, in murine macrophages, CB_2_R signaling has been shown to augment phagocytosis of zymosan particles, primarily through the activation of the Dectin-1 receptor pathway (95). Interestingly, 2-arachidonoylglycerol selectively enhances macrophage phagocytosis of zymosan particles while remaining inactive toward latex beads, *Escherichia coli*, *Staphylococcus aureus*, or apoptotic Jurkat cells (95). This effect, orchestrated by CB_1_R and CB_2_R, aligns with findings observed with CP55 940, a dual CB_1_R and CB_2_R agonist, where CP55 940 increased phagocytosis of the zymosan particles as well (95). Moreover, 2-AG has demonstrated the capacity to augment the phagocytic ability of avian HD11 macrophages toward zymosan particles (36). Conflicting perspectives emerge, with some studies suggesting that CB_2_R is responsible for enhancing phagocytosis (95), while another study highlights that, in murine macrophages, only CB_1_R (and not CB_2_R) activation enhances phagocytosis of zymosan particles (96). Moreover, CB_1_R activation triggers an upregulation of CB_1_R expression, hinting at a potential positive feedback loop sustaining macrophage phagocytic activity (96). In conclusion, the precise role of the ECS in the process of phagocytosis remains elusive and might be context dependent.

### Function of ECS in macrophage polarization

An indispensable function within the innate immune system lies in the capacity of macrophages to execute diverse functions regulated by alterations in their phenotype—a phenomenon termed polarization. The modulation of macrophage and microglia polarization by ECS has been addressed in various studies, revealing nuanced influences on macrophage behavior across diverse physiological contexts (Table 1).

**TABLE 1:** Impact of CB receptor activation on macrophage polarization in different disease models

Disease	Effect of CB1R signaling	Effect of CB2R signaling	Other treatments	Reference
Traumatic brain Injury (TBI)		Activation of CB2R using GP1a reduces neuroinflammation, promoting M2 polarization		(97)
Colorectal cancer	Activation of CB1R inhibits cancer cell proliferation, migration, and invasion and inhibits M2 polarization			(98)
Myofiber regeneration		Mice lacking CB2R (CB2R-KO) characterized by increased infiltration of M1 macrophages decrease in M2 macrophages		(99)
Cystic fibrosis (CF)		CB2R agonist downregulates M1 macrophage polarization and does not fully restore anti-inflammatory M2 macrophage polarization		(100)
Post-traumatic osteoarthritis		Activating CB2R shifts immune cell polarization away from pro-inflammatory states in mouse macrophages		(101)
Paraquat (PQ)-induced lung injury			WIN 55,212-2 increases the M2 macrophage numbers and reduces lung fibrosis	(102)
Liver fibrosis	CB1R blockade reduces M1-type bone marrow-derived monocytes/ macrophages			(103)
Alcoholic liver disease		CB2R activation using JWH-133 agonist reduces the expression of pro-inflammatory M1 genes in Kupffer cells without affecting the anti-inflammatory M2 profile		(104)
Spinal cord injury (SCI)		CB2R activation promotes M2 microglia differentiation, reduces pro-inflammatory cytokines		(105)
Acute liver failure		CB2R activation using JWH-133 agonist leads to macrophage polarization toward the M2 state while suppressing pro-inflammatory responses via miR-145		(106)
LPS-induced inflammation			WIN 55,212–2 impairs pro-inflammatory M1 polarization in human macrophages and inhibits cytokine production and inflammasome activation	(107)

Beginning with the CB_2_R, its significant role in promoting M2 macrophage polarization has been documented. For instance, examining the impact of CB_2_R deletion in a mouse skeletal muscle ischemia-reperfusion (IR) injury model revealed an increased infiltration of M1 macrophages and a concurrent decrease in M2 macrophages in CB_2_R-KO mice compared with wild-type counterparts, a finding corroborated in *in vitro* studies (99, 108–110). The promotion of M1 polarization and the inhibition of M2 polarization in CB_2_R-KO macrophages impeded the differentiation of myoblasts (99). Another study investigated the role of CB_2_R in macrophage polarization by using an agonist, lenabasum, that activated CB_2_R. In a CF model, lenabasum effectively reduced pro-inflammatory M1 macrophage polarization and cytokine secretion while enhancing phagocytic activity in CF macrophages (100, 111, 112). Additionally, studies in post-traumatic osteoarthritis (112), alcoholic liver disease (104), SCI (105), acute liver failure (106), and traumatic brain injury (TBI) (97, 113, 114)SCI (105) have all demonstrated the potential of CB_2_R activation to reduce inflammation and increase the presence of M2 macrophages and/or decrease M1 macrophages.

Conversely, CB_1_R appears to have an opposing role in macrophage polarization. In colorectal cancer, CB_1_R activation inhibited the differentiation of M2 macrophages, concomitant with reduced cancer cell proliferation, migration, and invasion. Conversely, CB_1_R inhibition had the opposite effects, suggesting CB_1_R as a potential therapeutic target in colorectal cancer (98). A similar trend emerged in liver fibrosis, where CB_1_R blockade led to a decrease in the number of M1 monocytes/macrophages (103).

Interestingly, a chemical treatment of cells with WIN 55212-2 increased the presence of M2 macrophages and reduced inflammation (102, 107). WIN 55212-2 is a CB receptor agonist that acts on cannabinoid receptors, CB_1_R and CB_2_R with Ki values 62.3 and 3.3 nM, respectively; therefore, its effects could be attributed to either of these receptors. In a study using a PQ-induced mouse model of lung injury, WIN 55,212-2 demonstrated dose-dependent protection against PQ-induced mortality. This treatment reduced inflammation fluid, lowered pro-inflammatory cells in bronchoalveolar lavage fluid, and increased the presence of M2 macrophages. WIN 55212-2 also improved lung histology, reduced fibrosis formation, and downregulated key fibrosis-related genes (102). In another study that used LPS treatment, WIN55,212-2 was shown to induce tolerogenic human monocyte-derived dendritic cells (WIN-hmoDCs) that are less responsive to LPS stimulation and proficient at priming regulatory T cells (Tregs) (97). In a study that used LPS treatment, WIN55,212-2 was shown to induce tolerogenic human monocyte-derived dendritic cells (WIN-hmoDCs) that are less responsive to LPS stimulation and proficient at priming regulatory T cells (Tregs) (107). Additionally, WIN55,212-2 impaired pro-inflammatory polarization in human macrophages, inhibiting cytokine production and inflammasome activation and preventing pyroptotic cell death. Mechanistically, WIN55,212-2 induces metabolic and epigenetic changes in macrophages by decreasing LPS-induced mTORC1 signaling, glycolysis commitment, and histone marks on pro-inflammatory cytokine promoters (97). All these findings highlight the potential of WIN 55212-2 as a modulator of macrophage phenotype and as a potential therapeutic agent in diverse inflammatory conditions.

In conclusion, the ECS influences macrophage polarization, with CB_2_R activation favoring anti-inflammatory M2 macrophages, while CB_1_R activation opposes M2 differentiation. The cannabinoid receptor agonist WIN 55212-2 emerges as a potent modulator of macrophage phenotype, demonstrating a consistent ability to enhance M2 presence, alleviate inflammation, and potentially serve as a therapeutic agent for diverse inflammatory conditions.

## THE IMPACT OF ECS AND CANNABINOIDS ON BACTERIAL INFECTIONS

Given the critical role of the ECS and cannabinoids on key innate immune system functions, particularly macrophage polarization, it is reasonable to assume that ECS significantly affects the host antimicrobial defense. Furthermore, the direct impact of cannabinoids on bacteria introduces an additional layer of complexity, highlighting the intricate interplay between the ECS and the host’s defense against bacterial infections. Here, we will review both the direct and host-directed effects of cannabinoids on bacterial pathogens.

### The direct impact of cannabinoids on the pathogens

The antimicrobial efficacy of *Cannabis sativa* extracts has been a subject of scientific examination since the 1950s, with initial investigations suggesting their antibacterial potential (115). Historically, various extraction methods have been employed to isolate *Cannabis sativa* extracts. Nevertheless, a significant breakthrough occurred in 1976 when specific cannabinoids, ∆9-THC, and cannabidiol (CBD), were identified for their unique bacteriostatic and bactericidal properties, showcasing notable efficacy against Gram-positive pathogens. In the early studies following this milestone, Δ9-THC and CBD demonstrated substantial effectiveness against various Gram-positive bacteria, including *Staphylococcus aureus*, *Streptococcus pyogenes*, *Streptococcus milleri*, and *Streptococcus faecalis*. However, their efficacy was limited when tested against Gram-negative bacteria such as *E. coli*, *Salmonella* Typhi, or *Proteus vulgaris* (116).

Subsequent studies corroborated these findings for various bacteria and cannabinoids (Table 2). For instance, pure CBD and major terpenes found in hemp essential oils (α-pinene, β-pinene, β-myrcene, α-terpinolene, and β-caryophyllene) demonstrated antibacterial activity against the various bacteria tested. In particular, CBD and monoterpenes exhibited notable antibiotic activity against *Listeria* and *Enterococcus* strains (117). Similarly, another study highlighted the potent antibacterial effects of cannabichromenic acid (CBCA), CBD, cannabichromene, cannabigerol, and Δ9-THC against Gram-positive antibiotic-resistant bacterial species, of *S. aureus*, and specifically also methicillin-resistant strains (118). In fact, CBCA showed potent and faster bactericidal activity than vancomycin, the current MRSA antibiotic. CBCA’s efficacy remained consistent across various conditions, maintaining mammalian cell viability (119). CBD’s efficacy extended to highly resistant pathogens such as *Staphylococcus aureus*, *Streptococcus pneumoniae*, and *Clostridioides difficile*. Importantly, CBD demonstrated robust activity against biofilms without inducing bacterial resistance after repeated exposure to this compound, positioning it as a promising candidate for further exploration as an antibiotic (120). Additionally, CBD exhibited the capability to eliminate a subset of Gram-negative bacteria, including the urgent threat pathogen *Neisseria gonorrhoeae* but also *Neisseria meningitidis*, *Moraxella catarrhalis*, and *Legionella pneumophila*. However, CBD was found to be inactive on its own against *Acinetobacter baumannii*, *Escherichia coli*, *Klebsiella pneumoniae*, *Pseudomonas aeruginosa*, *Stenotrophomonas maltophilia*, *Burkholderia cepacian*, *Proteus mirabilis*, *Salmonella* Typhimurium, *Shigella dysenteriae*, or *Haemophilus influenzae* (120).

**TABLE 2:** Direct impact of cannabinoids on pathogens^*a*^

Treatment type	Bacterial infection type	Effect on infections	Reference
Δ9-THC, CBD	*Staphylococcus aureus*, *Streptococcus pyogenes*, *Streptococcus milleri*, and *Streptococcus faecalis*	Antibacterial activity	(116)
CBCA, CBD, CBC, CBG, and Δ9-THC	*S. aureus*	Antibacterial activity	(118)
CBD, α-pinene, β-pinene, β-myrcene, α-terpinolene, and β-caryophyllene	*Listeria* and *Enterococcus*	Antibacterial activity	(117)
CBD	*Staphylococcus aureus*, *Streptococcus pneumoniae*, and *Clostridioides difficile*	Antibacterial activity, activity against biofilms	(120)
CBCA	*Staphylococcus aureus* (MRSA)	Bactericidal activity mediated by degrading bacterial membranes and altering the nucleoid	(119)
CBD	*Staphylococcus* species, *Listeria monocytogenes*, and *Enterococcus faecalis*	Enhancing bacitracin’s efficacy	(121)
CBD, Δ9-THC, and CBN	*Staphylococcus aureus* (MRSA)	Antibiotic activity, particularly in conjunction with methicillin	(122)
CBD	*E. coli*	Synergistic activities with erythromycin, rifampicin, and vancomycin	(123)
CBD	*Salmonella* Typhimurium	Synergistic activities with ampicillin, kanamycin, and polymyxin B	(124)
CBD	*Neisseria gonorrhoeae*, *Neisseria meningitidis*, *Moraxella catarrhalis*, and *Legionella pneumophila*	Antibacterial activity	(120)
CBD	*S. aureus* subsp. *aureus* Rosenbach	Synergistic activities with kanamycin	(123)
CBD	*Klebsiella pneumoniae*, *Escherichia coli*, and *Acinetobacter baumannii*	Synergistic activities with polymyxin B	(125)
HU-210	*Vibrio harveyi*	Negative impact on AI-2 QS, reduced QS-mediated biofilm formation and swimming motility	(126)
CBG	*Streptococcus mutans*	Anti-bacterial and anti-biofilm activities, negative impact on quorum sensing	(127)
CBD	*E. coli*	Inhibition of the release of membrane vesicles from bacteria	(123)

^*a*^ Δ9-THC, delta-9-tetrahydrocannabinol; CBC, cannabichromene; CBD, cannabidiol; CBCA, cannabichromenic acid; CBG, cannabigerol; CBN, cannabinol.

Moreover, cannabinoids exhibit the potential to enhance the efficacy of classical antibiotics, presenting a promising avenue for combating antibiotic-resistant bacteria. For instance, CBD was identified as a compound against antibiotic-resistant pathogens enhancing bacitracin’s (BAC) efficacy by at least 64-fold against Gram-positive bacteria (*Staphylococcus* species, *Listeria monocytogenes,* and *Enterococcus faecalis*). However, CBD’s impact was limited in Gram-negative bacteria (121). This study also identified other specific effects of CBD-BAC combination, including morphological changes in *S. aureus* during cell division and reduced membrane potential. Although there were no observed changes in the cell wall composition, CBD and BAC-treated bacteria displayed a decreased rate of autolysis by unknown mechanisms (121). Similarly, another study explored the antimicrobial properties against *Staphylococcus aureus* (methicillin-resistant *Staphylococcus aureus;* MRSA) of cannabinoids, including CBD, Δ9-THC, and Cannabinol (CBN) in combination (or not) with methicillin. All three cannabinoids demonstrated antibiotic activity against MRSA, with Δ9-THC showing incomplete growth inhibition. Combining cannabinoids with methicillin resulted in a significant reduction (at least 87.5%) in the MIC of methicillin (122). The study considered the mechanistic aspects of the effects of cannabinoids on the MRSA proteome, revealing changes in cellular responses and alterations in pathways such as protein biosynthesis, energy metabolism, and stress response, which indicated complex action mechanism (122).

CBD’s influence extended beyond Gram-positive to Gram-negative bacteria. When combined with certain antibiotics, such as erythromycin, rifampicin, and vancomycin, CBD significantly enhanced bactericidal action against Gram-negative bacteria but had minimal effect on *E. coli* when used alone (123). This enhancement was confirmed in other studies against *Klebsiella pneumoniae* and *Acinetobacter baumannii* (125). CBD also exhibited synergy with ampicillin, kanamycin, and polymyxin B as co-therapy against *Salmonella* typhimurium, where the low dosages of CBD-antibiotic combinations effectively inhibited *Salmonella* Typhimurium growth (124). Moreover, CBD augmented the antibiotic effects of kanamycin in Gram-positive bacteria (123). Additionally, CBD was used in combination with another antibiotic, polymyxin B, as a therapeutic combination against Gram-negative bacteria, including polymyxin B-resistant strains. Low polymyxin B concentrations enabled CBD to exert antibacterial effects on *Klebsiella pneumoniae*, *Escherichia coli*, and *Acinetobacter baumannii*, even against polymyxin B-resistant strains. Specifically, CBD, along with polymyxin B, exhibited synergistic effects against polymyxin B-resistant *K. pneumoniae* (125). However, CBD was found to be inactive on its own against *A. baumannii*, *Escherichia coli*, *Klebsiella pneumoniae*, *Pseudomonas aeruginosa*, *Stenotrophomonas maltophilia*, *Burkholderia cepacian*, *Proteus mirabilis*, *Salmonella* Typhimurium, *Shigella dysenteriae*, or *Haemophilus influenzae* (125).

The exact mechanism behind the anti-microbial function of cannabinoids such as CBD is not completely clear. However, the primary mechanism underlying CBD’s antimicrobial function is likely membrane disruption, which has been shown in a couple of studies. One study specifically explored how CBD affects bacterial cells, focusing on *S. aureus* and *Bacillus subtilis*. Notably, lipid synthesis showed a reduction below the MIC for CBD, indicating potential membrane-based effects. The observed membrane depolarization in *S. aureus* provided additional evidence of membrane activity changed in CBD treatment. In summary, CBD likely disrupts bacterial cytoplasmic membranes, though a specific molecular target is yet to be determined (120). Microscopic analysis of bacteria treated with cannabinoids also suggested that in the case of CBCA, the mode of action involves degrading bacterial membranes and altering the nucleoid (119). Interesting conclusions could also be drawn from studies on Gram-negative bacteria incubated with CBD. The limited effectiveness of CBD against most Gram-negative bacteria was attributed to the presence of the outer membrane and lipopolysaccharide. When using membrane-disrupting drugs or LPS-deficient bacteria, an increased susceptibility of Gram-negative bacteria to CBD was observed (125). Finally, a proteomic study that explored the antimicrobial properties of cannabinoids against *Staphylococcus aureus* (MRSA) of cannabinoids, including CBD, Δ9-THC, and CBN in combination (or not) with methicillin. All three cannabinoids demonstrated antibiotic activity against MRSA, with Δ9-THC showing incomplete growth inhibition. Combining cannabinoids with methicillin resulted in a significant reduction (at least 87.5%) in the MIC of methicillin (122).

Moving beyond traditional antimicrobial functions, cannabinoids intricately modulate specific aspects of bacterial physiology. In *Vibrio harveyi*—a fish pathogen (128), the synthetic cannabinoid HU-210 negatively impacted the autoinducer-2 (AI-2) pathway, reducing quorum sensing (QS)-mediated biofilm formation and swimming motility. HU-210 inhibited virulence factor production without affecting bacterial growth, suggesting potential applications as anti-QS agents in other bacteria (126). Given the universal nature of AI-2 as an autoinducer, the findings suggest potential applications for synthetic cannabinoids as anti-QS agents in other bacteria. Similarly, in *Streptococcus mutans*, the non-psychoactive compound cannabigerol (CBG) exhibited anti-bacterial and anti-biofilm activities, influencing the ComCDE and LuxS QS systems, reducing AI-2 production and influencing the crosstalk between ComCDE and LuxS pathways. CBG’s effects were demonstrated through increased susceptibility in knockout strains and alterations in gene expression, establishing CBG as a potential anti-QS compound (127).

Furthermore, cannabinoids also affect the generation or release of bacterial membrane vesicles (MVs), which are important structures involved in cell communication and host-pathogen interactions (129). One study found that CBD strongly inhibits the release of MVs from Gram-negative bacteria (*E. coli*) but has minimal effects on Gram-positive bacteria (*S. aureus*) (123).

In conclusion, cannabinoid interactions with bacterial infections yield diverse outcomes. While some studies suggest positive effects, conflicting findings highlight the intricate nature of these interactions, necessitating further research for a comprehensive understanding and cautious therapeutic applications.

### Role of cannabinoids in cellular infections with bacterial pathogens

In addition to their direct impact on bacteria, cannabinoids also influence host immune cells and other cellular components, resulting in complex and sometimes unpredictable consequences for the host’s defense against infections. While cannabinoids frequently exhibit antimicrobial properties, their ability to influence immune cell phenotypes may present challenges in resolving an infection.

While the role of CB_1_R signaling in macrophage function remains unresolved, the stimulation of CB_2_R provides a notable example frequently associated with promoting M2 macrophage polarization, as supported by several studies (99–101, 103, 104, 106). This M2 polarization is favored by certain bacterial pathogens, while in other cases, it is undesirable. Some bacteria, such as *Listeria* (130) and *Chlamydia* (131) tend to promote an M2 phenotype, whereas others, like *Ehrlichia* (99) prefer an M1 macrophage polarization. Intriguingly, *Salmonella* (132–134) appears to selectively regulate polarization toward either M1 or M2 phenotypes, depending on its infection stage. Therefore, regulation of M1/M2 polarization by cannabinoid receptor signaling is anticipated to have effects on the clearance of various bacteria differently.

In the context of *Legionella pneumophila* infection, the administration of Δ9-THC resulted in fatal acute collapse, intricately linked to a cytokine-mediated shock-like response. This response was characterized by elevated blood levels of TNF-α and IL-6. Remarkably, the protective effect of this cannabinoid was observed when mice were treated with antibodies against these cytokines, particularly anti-IL-6 treatment (135). Another study investigating Δ9-THC treatment in the same context revealed a shift from Th1 to Th2 immunity, mediated by both CB_1_R and CB_2_R. This modulation involved CB1R suppressing IL-12Rβ2 and CB_2_R enhancing GATA-3, a crucial transcription factor in Th2 cells. Additionally, Δ9-THC influenced cytokine production, including IFN-γ and IL-4, with CB_1_R and CB_2_R playing distinct roles in mediating these effects. Regulatory factors, such as Notch ligand Delta4 and Jagged1, were also influenced by Δ9-THC in a CB_2_R-dependent manner, concurrently elevating NFκB p65 activity in the spleen (136). It is worth noting that another cannabinoid, CBD, demonstrated antibacterial activity against *Legionella pneumophila* when tested *in vitro* (120).

Moving on to infections caused by *Listeria monocytogenes*, Δ9-THC, and marijuana extract exhibited an impact on host resistance, revealing immunosuppressive effects that significantly diminished the host’s ability to resist the infection. Notably, Δ9-THC displayed a dose-dependent negative outcome during *Listeria monocytogenes* infection, raising concerns about the potential public health implications of decreased host resistance resulting from marijuana extract and its cannabinoids (137). Interestingly, other cannabinoids, including CBD, α-pinene, β-pinene, β-myrcene, α-terpinolene, and β-caryophyllene, demonstrated antibacterial activity against the same bacterium *in vitro* (117). This again emphasizes the importance of assessing cannabinoid efficacy both *in vitro* and *in vivo*. In terms of oral pathogens, cannabinoids, including CBD, CBN, and Δ9-THC, were examined for their effects on three oral pathogens (*Porphyromonas gingivalis*, *Filifactor alocis*, and *Treponema denticola*) and the immune system. These cannabinoids were found to suppress pro-inflammatory cytokines and enhance anti-inflammatory cytokines in human innate cells. However, higher doses compromised cell viability and inhibited the growth of some oral bacteria. The study suggested that environmental cannabinoids might contribute to periodontitis through direct bacterial toxicity, compromised cell vitality, and suppressed immune responses via the CB_2_R/PI3K pathway (138).

In contrast, in some instances, cannabinoids exhibited a protective effect. For example, the efficacy of HU-211 was evaluated in combination with antimicrobial therapy for reducing brain damage in experimental pneumococcal meningitis. Rats infected with *Streptococcus pneumoniae* were treated with saline, ceftriaxone alone, or a combination of ceftriaxone and HU-211. The results demonstrated a significant reduction in brain edema and blood-brain barrier impairment 48 hours after infection in rats receiving ceftriaxone-HU-211 compared with other treatment groups (139). HU-211 diverges in its mode of action by not functioning as a cannabinoid receptor agonist; instead, it operates as an NMDA antagonist (140).

Further exploration into the effects of eCBs on the host response to infections revealed interesting findings. The ABHD6 and FAAH, responsible for the breakdown of 2-AG, were identified in chicken macrophages by chemical proteomics (36). Infection of these macrophages by *Salmonella* Typhimurium led to a decrease in the activity of these enzymes (36), therefore suggesting stabilization of 2-AG during infection. Moreover, 2-AG, a primary endogenous ligand for the CB_2_R, led to improved phagocytosis of zymosan particles (36). Subsequent research showed promise in using elevated 2-AG levels to defend against infections caused by intracellular bacteria, including *E. coli*, *Citrobacter rodentium*, and *Salmonella* Typhimurium (141). This protection was achieved by inhibiting critical virulence programs, particularly the QseC system essential for bacterial infection, and antagonizing the type three secretion system. Moreover, transgenic mice engineered to produce elevated 2-AG levels exhibited increased survival rates upon *Salmonella* infection, suggesting a potential role for CBs in bacterial infection control (141).

CB_2_R was explored in regulating the neutrophil migration—a key factor in inflammation (142). Endocannabinoids, specifically N-acyl ethanolamine type, are exported by P-glycoprotein in intestinal cells, contributing to immune balance. In this context, the absence of CB_2_R intensified inflammatory responses and neutrophil infiltration. To investigate the function of the CB_2_R further, neutrophils isolated from both wild-type and CB_2_R-deficient mice were tested for migration across epithelial layers infected with *Salmonella*, in the presence of Activity Modulating Epithelial Neutrophil Discourse (AMEND) containing endocannabinoids. The experiments demonstrated that CB_2_R signaling was crucial for AMEND’s inhibitory effect on neutrophil migration. While AMEND was less effective without CB_2_R, higher concentrations still impeded neutrophil migration, suggesting the involvement of additional receptors (142). Another study revealed that neutrophils treated with 2-AG eCB released α-defensins and LL-37, which have antimicrobial properties and recruit leukocytes. Supernatant from 2-AG-stimulated neutrophils was observed to diminish the growth of *E. coli* and *Staphylococcus aureus*, indicating a potential mechanism through which host immune cells may utilize cannabinoids to promote bacterial clearance (143). These two studies illustrate the control of neutrophile responses by cannabinoid signaling during bacterial infections.

Examining the effects of cannabinoid receptor activation on bacterial infection, a synthetic CB (JWH133, CB_2_R agonist) was investigated in *Pseudomonas aeruginosa* infection causing acute lung injury and acute respiratory distress syndrome (144). Mice treated with JWH133 exhibited favorable clinical outcomes, including a significant decrease in pro-inflammatory cytokines, decreased bacterial load in the lungs, reduced neutrophil activation, and a decrease in NF-κB and NLRP3 inflammasome activation. The effects of cannabinoid treatment were abolished when CB2R was knocked out, indicating that the activation of this specific receptor is responsible for the improved outcomes (144).

Another interesting facet of the eCB system’s effect on *Staphylococcus aureus* infection was explored in the house musk shrew, a model to evaluate the emetic effects of Staphylococcal enterotoxins produced by these bacteria. The study demonstrated that the activation of CB1R by WIN 55,212-2 agonist could mitigate SEA-induced emesis by reducing 5-hydroxytryptamine (5-HT) release in the intestine. Furthermore, a selective CB_1_R antagonist, AM 251, when given before treatment with WIN 55,212-2 and SEA, reversed the effect of WIN 55,212-2 on this model (145). In a different infection model for the same pathogen, CBD demonstrated effective *in vivo* efficacy in a topical skin *Staphylococcus aureus* infection model. However, the limited systemic activity of CBD, attributed to high serum binding, poses a challenge. The study proposed that modifying CBD’s core structure could enhance its systemic effectiveness (120).

Another investigation focused on cannabinoid’s effect on macrophages combating *Brucella suis*, an intracellular Gram-negative bacterium. The CB_1_R antagonist, SR141716A, demonstrated a pronounced inhibition of *Brucella* multiplication within macrophages, emphasizing the role of CB_1_R in activating macrophages against this bacterium and augmenting their antimicrobial properties (146). Specifically, the impediment of the elevation of cAMP induced by *Brucella* was attributed to the action of SR141716A. This observation marked the initiation of discerning a second messenger induction during the early stages of macrophage infection by *Brucella*, thereby establishing a direct association with bacterial virulence.

Finally, a study explored the association between cannabis use and *Helicobacter pylori* infection in a representative community sample. Using data from the National Health and Nutrition Examination Survey III, the study categorized cannabis usage into ever-use, cumulative lifetime use, and recent use. Interestingly, cannabis use was associated with a decreased risk of *H. pylori*, and this association persisted even after adjusting for demographics and comorbidities. Additionally, individuals with higher cumulative lifetime cannabis use (>10 times) showed a further decreased risk of *H. pylori* compared with those with lower lifetime use. The findings suggest a potential association between recreational cannabis use and a reduced risk of *H. pylori*, although the reason for this effect is completely unknown (147).

In summary, the impact of cannabinoids on bacterial infections is complex, showing both positive and negative effects in various studies. Positive outcomes include the therapeutic potential of cannabinoids in conditions like pneumococcal meningitis (139). Additionally, cannabinoids such as CBD (120) or synthetic cannabinoids (144–146) have demonstrated antibacterial properties, influencing the immune response or improving clinical outcomes in some cases. However, contrasting findings reveal adverse effects, such as a fatal collapse in *Legionella pneumophila* infection induced by Δ9-THC (135, 136) and immunosuppression during *Listeria monocytogenes* infection by Δ9-THC and marijuana extract (137). Therefore, despite some studies highlighting the positive impact of cannabinoid treatment (Table 3) [reviewed in reference (148)], the negative impact of cannabinoids in other infections or systems suggests that the effects of cannabinoids and cannabinoid receptor signaling require systematic study. This is primarily due to the complexities inherent in the interactions between cannabinoids, bacteria, and the immune system. Ideally, cannabinoid effects on the host response to infection should be explored with a focus on receptor-specific effects.

**TABLE 3:** Effects of phytocannabinoids, cannabinoids, and cannabinoid receptor agonists on bacterial infections

Treatment type	Bacterial infection type	Effect on infections	Reference
Δ9-THC	*Legionella pneumophila* infection	Fatal acute collapse, cytokine-mediated shock-like response with elevated TNF-α and IL-6; shift from Th1 to Th2 immunity, modulation involving CB1R and CB2R, influence on cytokine production	(135, 136)
Δ9-THC, marijuana extract	*Listeria monocytogenes* infection	Immunosuppressive effects, decreasing host resistance	(137)
CBD, CBN, Δ9-THC	Oral pathogens (*Porphyromonas gingivalis*, *Filifactor alocis*, *Treponema denticola*)	Suppression of pro-inflammatory cytokines, enhanced anti-inflammatory cytokines, compromised cell viability	(138)
HU-211	Experimental pneumococcal meningitis (*Streptococcus pneumoniae*)	Reduction in brain edema and blood-brain barrier impairment	(139)
Elevated 2-AG levels	*Escherichia coli*, *Citrobacter rodentium*, *Salmonella enterica*	Improved phagocytosis, increased survival rates	(141)
2-AG supplementation	*Escherichia coli* and *Staphylococcus aureus*	Supernatant from 2-AG-stimulated neutrophils inhibited bacterial growth	(143)
SR141716A (CB1R antagonist)	*Brucella suis* within macrophages	Inhibition of *Brucella* multiplication, activation of macrophages	(146)
JWH133 (CB2R agonist)	*Pseudomonas aeruginosa*	Decreased pro-inflammatory cytokines, reduced bacterial load, improved clinical outcomes	(144)
CBD	Topical skin *Staphylococcus aureus* infection model	Effective *in vivo* efficacy, limited systemic activity	(120)
WIN 55,212-2 (CB2R and CB1R agonist)	*Staphylococcus aureus*-induced emesis in house musk shrew	Mitigation of SEA-induced emesis, reduction in 5-HT release in the intestine	(145)
Cannabis use	*Helicobacter pylori* infection	Decreased risk of *H. pylori,* potential association with reduced risk	(147)

### Effect of ECS on sepsis

The ECS, particularly CB receptors, emerges as a crucial regulator in the immune response to sepsis—a life-threatening condition, often resulting from dysregulated immune reactions (65, 149) triggered by bacterial infections.

In a well-established murine sepsis model involving cecal ligation and puncture (CLP), researchers explored CB_2_R modulation’s impact on the immune response. CB2R-deficient mice displayed reduced survival rates, heightened inflammation (elevated serum IL-6 levels), and increased lung injury compared with wild-type mice. A CB2R agonist improved survival rates and mitigated inflammation, bacteremia, and lung damage in septic mice, suggesting CB_2_R as a potential therapeutic target for sepsis (150). In another study using the CLP model, mice with CB_2_R knocked out in CD4+ T cells showed improved survival and increased IL-10 production upon CB_2_R activation, indicating that CB_2_R activation in CD4+ T cells may enhance sepsis susceptibility by inhibiting IL-10 production. Clinical findings also revealed elevated CB_2_R levels in septic patients, correlating with lower IL-10 levels (151). However, contrasting results emerged in another study deploying the same polymicrobial sepsis model via CLP methodology, where CB_2_R-KO mice had reduced sepsis-related mortality and bacterial translocation into the bloodstream. Moreover, the CB_2_R-KO animals had also lessened kidney and muscle damage, suppressed the activation of NF-κB in the spleen, and decreased the production of specific immune-regulating molecules such as IL-10, IL-6, and MIP-2 (152). It is worth noting that these studies diverged in their findings concerning survival, with markedly different mortality among wild-type mice in response to knockout of cannabinoid receptors. A comprehensive analysis of these mortality discrepancies within sepsis models involving CB_2_R-KO mice can shed light on the pivotal role of infection severity in determining survival. Using the CB_2_R to reduce inflammation in moderate sepsis models may hinder bacteria clearance, increasing mortality. Conversely, in severe sepsis, heightened inflammation worsens damage and mortality, suggesting CB_2_R targeting for severe infections.

Examining the effects of ECS signaling on severe microbial illnesses like sepsis warrants an exploration of the CB_1_R. CB_1_R is mainly found in the central nervous system and is involved in functions like temperature regulation, pain perception, and motor control. These receptors are also present in vital organs like the liver, kidneys, pancreas, and cardiovascular cells. Studies in sepsis models have shown that CB_1_R activation can affect blood pressure, with CB_1_R antagonists increasing blood pressure in various sepsis models (153–155). Additionally, CB_1_R activation has been associated with changes in body temperature during sepsis, including hypothermia, linked to an increased risk of mortality. For example, a study employing the CB_1_R antagonist rimonabant demonstrated reduced hypothermia in the context of LPS-induced systemic infection, highlighting CB_1_R’s role in regulating body temperature during infection (156). Moreover, CB_1_R was found to play a role in initiating fever and heightened pain sensitivity (hyperalgesia) in response to LPS in mice. When exposed to a low dose of LPS, normal mice experienced significant and sustained fever. In contrast, mice lacking CB_1_R (CB_1_R-KO) or those treated with a CB_1_R antagonist did not develop a fever, although they retained the ability to exhibit a quick increase in body temperature due to injection stress and TLR3 activation. The diminished response to LPS in CB_1_R-KO mice may be linked to reduced TLR4 receptors in peripheral immune cells (157).

Furthermore, cannabinoid treatments have been examined in animals in the context of inflammation. CBD was found to have complex effects on the immune system. Oral administration of CBD in mice increased inflammation induced by LPS, primarily involving neutrophils and monocyte populations present in the bronchoalveolar lavage fluid. Additionally, CBD upregulated the transcription of genes encoding pro-inflammatory proteins, including TNF-α, IL-5, IL-23, and GCS-F (158).

Lastly, beyond the roles of CB_1_R and CB_2_R in immune and physiological regulation, researchers have explored the indicators of altered eCB production as potential markers for sepsis severity. A study encompassing 106 hospitalized sepsis patients showed that patients with diminished AEA and 2-AG levels necessitated extended hospital stays and invasive mechanical ventilation (159).

In summary, research on murine sepsis models demonstrated the potential therapeutic value of CB_2_R modulation, with CB_2_R activation improving survival and mitigating inflammation while revealing complexities in its role within CD4+ T cells. Moreover, investigations into the CB_1_R influence on blood pressure, body temperature, fever, and pain sensitivity underscore its significance in severe microbial infections like sepsis. While some research groups have initiated investigations into the ECS role in sepsis, much of this territory remains uncharted. Urgently needed is further research to explore the potential of the ECS as a target for modulating host immune responses and improving outcomes in life-threatening sepsis.

### Role of CB signaling in microbiota alterations

The exploration of the eCB system’s role in energy homeostasis has extended into studies on fat intake and storage, key elements in the balance of energy within the body (160). The increased expression of cannabinoid receptors in the gastrointestinal tract hints at a significant impact on the host’s gut microbiome, prompting a deeper investigation into how alterations in microbial composition influence eCB synthesis and receptor expression.

Initial investigations into this domain have emphasized the direct impact of gut microbiota alterations on the cannabinoid system, influencing both the production of cannabinoids and the expression of their receptors. A quintessential illustration of this interaction is observed with *Lactobacillus acidophilus*. Administration of this probiotic strain leads to a notable upregulation of CB_2_R transcript expression in mice (161), indicating that specific microbial strains could beneficially influence CB_2_R expression and thus contribute to gut health regulation. This relationship between microbiota changes and cannabinoid receptor and endocannabinoid level regulation is substantiated by research linking the ECS to microbial imbalances and metabolic disorders, such as obesity and diabetes. For example, the administration of *Akkermansia muciniphila* into the gastrointestinal tract of diet-induced obese mice demonstrated significant restoration of gut barrier functions, but it also caused an increase in intestinal endocannabinoids, establishing a possible link between *A. muciniphila* treatment and enhanced intestinal health (162). Conversely, another study exploring dietary capsaicin’s impact on the gut microbiome revealed a reduction in LPS-producing bacteria in mice, which was associated with decreased CB_1_R expression in these animals (163).

The bidirectional nature of the microbiota-ECS relationship is illustrated by findings showing that eCB system changes can prompt shifts in microbiota composition. Studies comparing the gut microbiome and metabolome of individuals with inflammatory bowel disease (IBD) to healthy controls have found significant differences, with specific metabolites elevated in IBD patients (164). Notably, such metabolites like linoleoyl ethanolamide, palmitoylethanolamide, and N-oleoylethanolamine were found to be significantly elevated in IBD patients (164). Building on these discoveries, further research highlighted linoleoyl ethanolamide, a specific N-acylethanolamine (NAE), for its role in encouraging the growth of bacteria associated with IBD progression (165). Among NAEs, only AEA is recognized as a true endocannabinoid. This study demonstrated that NAEs could foster the growth of bacterial strains prevalent in IBD, such as *Escherichia coli*, while suppressing other bacteria, thus skewing the gut microbiome toward an IBD-like profile. Through metagenomic and metatranscriptomic analyses, the investigation revealed that NAEs induce shifts in microbial composition toward an IBD-like state, marked by an increase in Proteobacteria and a decrease in Bacteroidetes, alongside the upregulated expression of respiratory chain components, reflecting NAEs’ impact on bacterial metabolism. Importantly, this research identified NAEs as a class of endogenous signaling lipids capable of transforming the gut microbiome into an environment conducive to IBD (165).

Furthermore, the exploration of cannabinoids’ impacts on the microbiome has been extended through the application of HU-210, a synthetic cannabinoid known for its strong affinity for both CB_1_R and CB_2_R. This study revealed that HU-210 treatment led to an increase in plasma LPS levels, thereby shedding light on the ECS’ essential role in maintaining gut barrier integrity. Such findings emphasize the significance of ECS in sustaining the stability of the gut microbiome ecosystem (166). Building on this, a study involving Diet-Induced Obesity (DIO) models has shown the potential of CB_1_R blockers in effectively managing obesity and related metabolic conditions. The use of these blockers has been shown to induce beneficial shifts within the gut microbiota, notably increasing *Akkermansia muciniphila* populations while reducing those of Lachnospiraceae and Erysipelotrichaceae. This suggests that targeting the CB_1_R might offer a novel approach to obesity management and the improvement of associated metabolic disorders through the modulation of gut microbiota (167).

The endocannabinoid 2-AG is hydrolyzed by the enzyme MGLL and plays a role in intestinal dysfunction (168). MGLL knockout mice (MGLL-KO) were used to evaluate the relationship between intestinal dysfunction and alteration of the gut microbiome. MGLL-KO mice had a different gut microbiome compared with wild-type mice when given a high-fat diet. The MGLL-KO mice had lower levels of bacterium such as *Lactobacillales*, *Prevotellaceae*, *Coriobacteriaceae*, *Erysipelotrichaceae*, *Clostridium_XIVa*, and increased *Eubacteriaceae* (169). This phenomenon is most likely due to the change in free fatty acids in the system, providing additional nutrients to the more competitive bacterium.

The gastrointestinal receptor GPR119, which is engaged in energy homeostasis and metabolic processes, emerges as a nexus for both eCBs and microbial byproducts within the host’s microbiome (170). The ability of GPR119 to be activated by microbial byproducts akin to eCBs illuminates the gut microbiome’s capacity to generate molecules that influence host physiology in a manner reminiscent of eCB signaling, thereby emphasizing the profound interdependence of the host’s metabolic health and its microbiome.

Shifting the focus from metabolic health to autoimmune conditions, an intriguing study on experimental autoimmune encephalomyelitis (EAE)—a model for multiple sclerosis—examined the interplay between cannabinoid treatment and microbiome composition. Typically, EAE models are characterized by an overabundance of Gram-negative bacteria. Remarkably, treatment with Δ9-THC and CBD, both phytocannabinoids, resulted in a notable decrease in *Akkermansia muciniphila*, alongside reduced circulating LPS levels. This outcome not only highlights the potential of cannabinoids in fostering a healthier gut microbiome but also suggests a broader therapeutic relevance, especially in the context of autoimmune disorders (171).

Research on the ECS demonstrates its critical role in modulating the gut microbiome, highlighting how changes in microbial composition can affect eCB synthesis and receptor activity. These insights have profound implications for treating metabolic and inflammatory conditions. Furthermore, studies on synthetic CBs and the modulation of enzymes like MGLL underscore the therapeutic potential of targeting the gut microbiome to improve metabolic health.

## CONCLUSIONS

The cellular roles of the ECS often yield contrasting findings in the context of innate immune cells, the microbiome, and intracellular infections. Notably, CB_2_R signaling stands out as a pivotal influencer in these areas, impacting diverse aspects such as immune cell migration, morphological changes, autophagy, apoptosis, phagocytosis, and macrophage polarization.

The bidirectional relationship between the ECS and gut microbiota further highlights the interconnectedness of the physiological processes of the host and bacteria. Microbial dysbiosis can influence CB receptor expression, while CBs can also impact the composition and health of the gut microbiome. These findings open new avenues for exploring the role of ECS in metabolic health and inflammation.

Furthermore, the ECS involvement in infections caused by bacterial pathogens and polymicrobial sepsis models adds complexity to our understanding of its role in infections. While many studies suggest the potential of CBs in controlling host responses to bacterial infections, outcomes vary depending on the specific infection, cannabinoid, and cannabinoid receptor involved. Further research is essential to elucidate the mechanisms and clinical implications fully. The impact of synthetic CBs, eCBs, and phytocannabinoids on the clinical outcome and host response to infection likely needs to be studied individually for each pathogen. Furthermore, given the increasing use of cannabis and phytocannabinoids such as CBD for medical purposes (172), it is necessary to understand their potential effects on specific infections.

The potential therapeutic use of cannabinoids for infectious diseases remains unclear. However, several cannabinoids have received approval for medical treatments unrelated to infectious diseases. For instance, the CBD oral solution, known as Epidiolex, was approved by the US Food and Drug Administration (FDA) for treating seizures associated with Lennox-Gastaut syndrome, Dravet syndrome, and seizures linked to tuberous sclerosis complex (173). Additionally, THC and cannabidiol are combined as a buccal spray named Sativex, approved for neuropathic pain associated with multiple sclerosis and licensed in 25 countries (174). Dronabinol (Marinol, Syndros), acts as a synthetic THC approved by the FDA for combating chemotherapy-induced nausea (175) and stimulating appetite in individuals with AIDS (176). Nabilone, a synthetic analog of THC marketed as Cesamet, is FDA approved as an antiemetic in chemotherapy patients (177) and utilized off-label for spasticity-related pain in patients with upper motor neuron syndrome (178). Furthermore, Rimonabant, a selective CB_1_R receptor antagonist, was once available in Europe for obesity treatment but was withdrawn due to psychiatric side effects and was not approved in the United States (179).

In summary, the cellular roles of the ECS transcend its initial discovery and its established roles in maintaining homeostasis and regulating brain functions. This intricate system extends its influence to encompass immune regulation, bacterial infection responses, and the dynamics of the gut microbiota. As ongoing research unveils the profound intricacies of the ECS, it emerges as a promising target for therapeutic interventions across a broad spectrum of medical conditions, including bacterial infection, dysbiosis, and sepsis.
